# Minimally Invasive Medial Plating of Low-Energy Lisfranc Injuries: Preliminary Experience with Five Cases

**DOI:** 10.1155/2016/4861260

**Published:** 2016-05-31

**Authors:** Jorge Javier del Vecchio, Mauricio Ghioldi, Nicolás Raimondi, Manuel De Elias

**Affiliations:** ^1^Favaloro Foundation University Hospital, 461 Solis Street, 1st Floor, 1078 Ciudad Autónoma de Buenos Aires, Argentina; ^2^Austral University Hospital, 1500 Presidente Perón Avendia, 1629 Pilar, Argentina

## Abstract

Fracture dislocations involving the Lisfranc joint are rare; they represent only 0.2% of all the fractures. There is no consensus about the surgical management of these lesions in the medical literature. However, both anatomical reduction and tarsometatarsal stabilization are essential for a good outcome. In this clinical study, five consecutive patients with a diagnosis of Lisfranc low-energy lesion were treated with a novel surgical technique characterized by minimal osteosynthesis performed through a minimally invasive approach. According to the radiological criteria established, the joint reduction was* anatomical* in four patients,* almost anatomical* in one patient (#4), and* nonanatomical* in none of the patients. At the final follow-up, the AOFAS score for the midfoot was 96 points (range, 95–100). The mean score according to the VAS (Visual Analog Scale) at the end of the follow-up period was 1.4 points over 10 (range, 0–3). The surgical technique described in this clinical study is characterized by the use of implants with the utilization of a novel approach to reduce joint and soft tissue damage. We performed a closed reduction and minimally invasive stabilization with a bridge plate and a screw after achieving a closed anatomical reduction.

## 1. Introduction

Fracture dislocations involving the Lisfranc joint are rare; they represent only 0.2% of all the fractures [1]. Although most of the lesions occur after a high-energy trauma, such as motor vehicle accidents, work accidents, and falls from heights, they can also be associated with low-energy accidents, such as strains, particularly in athletes and elderly people [2]. In these cases, fracture dislocations may be mild and less severe than those caused by heavy trauma, but if they are not adequately managed they may be associated with significant morbidity [3]. Goossens and De Stoop [4], among others, estimate that almost 20% of the Lisfranc fracture dislocations go undiagnosed [3, 5]. Due to the high potential for chronic disability, both early diagnosis and anatomical reduction are essential [6–11].

There is no consensus about the surgical management of these lesions in the medical literature [12, 13]. However, both anatomical reduction and tarsometatarsal stabilization are essential for a good outcome [3, 14–16].

A large number of treatment options have been proposed for this type of fracture dislocations, such as fixation with percutaneous pegs and/or screws; other surgeons prefer open reduction and internal fixation (RAFI) [14, 16–18].

The objective of this case series is to study five consecutive patients with a diagnosis of Lisfranc low-energy lesion treated with a novel surgical technique characterized by minimal osteosynthesis performed through a minimally invasive approach; the focus will be the analysis of the clinical and radiological results.

## 2. Methods

All the patients included in the study signed a written informed consent; the study protocol was approved by the Teaching Committee of the Favaloro Foundation University Hospital.

### 2.1. Demographics

A prospective study was designed. In the period February–May 2014, 5 consecutive patients (4 females, one male; mean age, 42.4 (age range, 25–67)) were enrolled. The patients had a diagnosis of tarsometatarsal fracture dislocation caused by low-energy trauma. A closed reduction and minimal osteosynthesis were performed by MIS (minimally invasive surgery); a 2.7 mm bridge plate was implanted between the first cuneiform (C1) and the first metatarsal (M1), and a 3.0 mm cannulated screw was placed between C1 and the second metatarsal (M2). The patients were treated exclusively by the first author (Jorge Javier del Vecchio). The average follow-up was 19.4 months (range, 18–21 months). The patients were identified by search criteria in the Trauma Registry database. They were contacted via e-mail and/or telephone and were called for both clinical and radiographic results.

*Inclusion Criteria*

- Patients with a mature skeletal system at the time of the lesion.
- Low-energy trauma involving the medial column and medial tarsometatarsal joint.
- Pure ligament lesions or fractures associated with ligament lesions.

*Exclusion Criteria*

- History of previous surgery of the foot or ipsilateral ankle.
- Presence of active infection.
- Fractures in the ipsilateral concurrent lower limb.
- Chronic injuries (more than 6 weeks).
- Previous surgical management of the same lesion.
- Presence of comminuted fractures in the first three metatarsals suggestive of high impact trauma.

### 2.2. Radiological Assessment

Joints presenting parallelism between the medial border of the second metatarsal and the medial border of the second cuneiform in the anteroposterior projection were considered congruent. The lateral border of the third metatarsal and the lateral border of the lateral cuneiform must line up on the oblique view [19].

The tarsometatarsal angle was also used to assess the sagittal deformity. Apart from the static and the radiological assessment, stress images were obtained in patients with a clinical suspicion of a tarsometatarsal lesion in the static X-rays showing a congruous result. These images were obtained with fluoroscopy under analgesia, with pronation and simultaneous abduction maneuvers to detect diastasis or angulation [20].

The reduction was considered anatomical when the above was intact, almost anatomical if the displacement was equal to or over 2 mm, and nonanatomical if the distance was over 2 mm (or more than 15° talus-first metatarsal angulation) [3, 10, 21, 22].

Fractures were classified according to the system described by Myerson [3]:

- Type A: total incongruence.
- Type B: partial congruence.
- Type C: one divergence.

All the cases were classified as Type B2.

### 2.3. Clinical Assessment

The patients were assessed according to the American Assessment Scale of the Orthopaedic Foot and Ankle Society (AOFAS) of the midfoot set by Kitaoka et al. [23] for clinical assessment at least 18 months after surgery. Pain assessment was conducted by using the Analog Visual Scale. Subjective assessment was also conducted by an “ad hoc” questionnaire. The duration of the reconstructive procedures was recorded in minutes.

### 2.4. Surgical Technique

The patient is placed in the dorsal decubitus position with a small pillow at the level of the ipsilateral hip to neutralize the position of the limb. Only one dose of antibiotic is administered intravenously for infection prophylaxis. A tourniquet at 270 mm Hg at the supramalleolar region was used for the procedure. Image intensifier was necessary to control the reduction and position of the osteosynthesis.

First, any potential displacement or incongruence between the first and second cuneiform is detected. The first TMT joint is aligned after reducing the medial border of the first cuneiform in the medial border of the first metatarsal.

**Figure 1:**
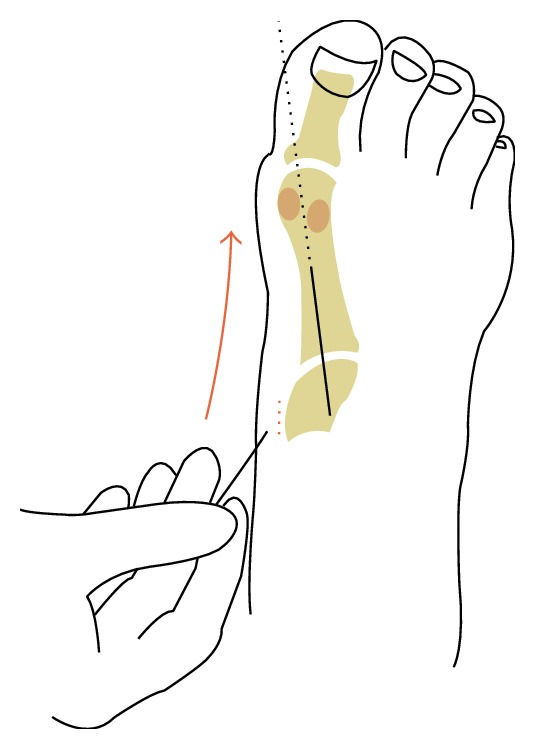
A 2.7 mm low-profile plate is slid minimally invasively through a proximal incision to achieve normal abduction of the medial column.

Although the preliminary fixation through the TMT joints may be performed with 1.5 mm Kirchner pegs, they may also cause intra-articular damage, mainly if several attempts are required. Later, a low-profile plate is placed in the dorsomedial region to prevent damaging the plantar-medial insertion of the anterior tibial tendon. The screws of the plate were placed bicortically to prevent damage of the joint surface, the intercuneiform joint, and the cuneiform-metatarsal joint. The first 15 mm portal was performed in the medial aspect of the first cuneiform bone. Then a 2.7 mm low-profile plate, locked and slightly premolded in varus, is inserted to achieve normal abduction of the medial column to then reach the medial metaphyseal region of the first metatarsal (Figure 1).

**Figure 2:**
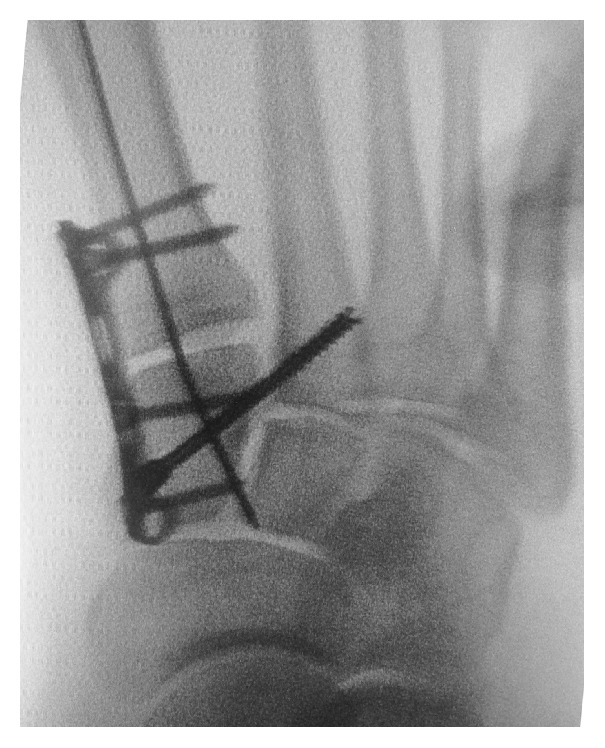
End fluoroscopic control prior to removal of the K wire.

The first screw is inserted. Then, the distal portion (second portal) is locked temporarily by means of a reduction instrument. Then, the sagittal orientation of the plate is checked so as to confirm plantar longitudinal arch reconstruction, by pivoting with the proximal screw. Once the position has been achieved and after checking that the C1-M1 joint is not compressed or no diastasis has occurred, either 1 or 2 screws may be placed on each joint side. Then, C1-M2 is reduced using bone clamps for bone reduction so as to close any opening between the first two columns. In order to stabilize the columns, a cannulated and partially threaded screw is inserted avoiding excessive joint compression (Figure 2).

**Figure 3:**
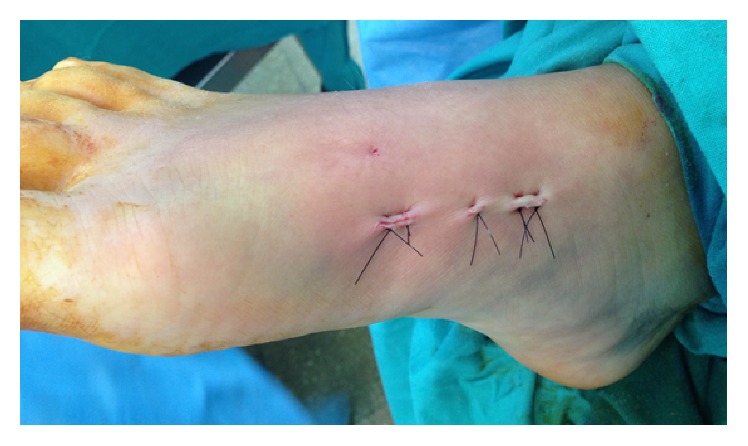
Clinical result after MIS Lisfranc bridge plating.

**Figure 4:**
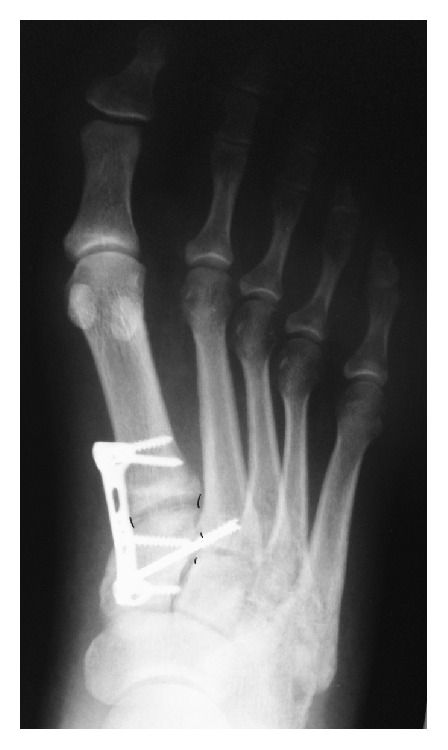
Initial postop. Rx showing congruency of the first two columns.

Thus, an anatomical position of the medial and middle columns is obtained without tarsometatarsal joint involvement (Figures 3 and 4). Finally the K wire is removed.

### 2.5. Postoperative Protocol

 The postoperative protocol was non-weight-bearing for six weeks. Afterwards, weight bearing was started with the help of a removable Walker boot, and internal fixation removal was scheduled for 4 months after surgery. Follow-up images were studied and clinical assessment was conducted by orthopedic surgeons specialized in the foot and ankle considering the radiographic parameters described.

## 3. Results

According to the radiological criteria established, the joint reduction was* anatomical* in four patients,* almost anatomical* in one patient (#4), and* nonanatomical* in none of the patients. At the final follow-up, the AOFAS score for the midfoot was 96 points (range, 95–100). The patients substracted points more often due to mild pain, limitations in recreational activities, and the impossibility of wearing fashionable shoes. As for subjective satisfaction, all the patients reported they were pleased with the procedure and the results obtained. The mean score according to the VAS (Visual Analog Scale) at the end of the follow-up period was 1.4 points over 10 (range, 0–3). All the patients achieved complete weight load at an average of 42.4 days (40–46) considering the initial premise of no body weight bearing for 6 weeks. None of the patients required assistance or braces (for the foot, splints or orthopedic shoes, etc.) after restarting their daily activities. The osteosynthesis material was removed in all patients as scheduled after an average of 17 weeks (range, 16–18). No complications (soft tissue or bone infection, delayed healing, chronic pain, etc.) were recorded.

Average surgical time for the procedures was 47.4 minutes (range, 40–52); see Table 1.

## 4. Discussion

Lisfranc fracture dislocations are still a difficult condition to manage for orthopedic surgeons and may be associated with severe morbidity [24]. Patients may experience chronic pain and functional loss due to arthrosis, deformity, residual instability, and associated soft tissue lesions [25]. Radiological diagnoses of the large fracture dislocations are obvious, but subtle lesions tend to be subdiagnosed. X-rays with weight load are the most widely used tool to diagnose these lesions [26].

However, the authors believe that they are not enough to assess patients with low-energy hidden lesions (negative static X-rays), for some lesions may exhibit dislocation or latent diastasis that can only be detected in stress X-rays. In our series, the lesions were seen in the radiological studies, and therefore complementary studies (MRI, CT scan, etc.) were not necessary. Lesions were classified according to the classification of Myerson et al. [22]. For a radiological classification to be useful, the classification system must be coherent, reliable, and easy to interpret. Mahmoud et al. [27] investigated intraobserver reliability of the Hardcastle classification system, modified by Myerson among several observers for a two-week period, and found significant agreement. The authors suggest that this classification system has proved to be a reliable tool for the assessment of Lisfranc lesions and may be used in clinical evaluation studies for it offers the standard terminology used by physicians.

Similar criteria to those of other authors were used for the clinical assessment [21, 28, 29], supporting the fact that the parameters set by Myerson et al. of more than 2 mm of residual tarsometatarsal displacement or more than 15° of persistent angulation between the talus and the first metatarsal after closed reduction attempts are useful to follow an open procedure. As described, a minimally invasive technique was used and so the conversion to open surgery was not necessary in any of the cases.

Anatomical reduction associated with stable internal fixation has become the treatment of choice for Lisfranc fracture dislocations [3, 8, 22, 26, 30]. However, there is controversy as to how to achieve this result [3]. Although the attending orthopedic surgeon can not control the scope of the damage caused by the initial lesion, in particular in high-energy trauma historically associated with poorer outcomes, they may minimize additional damage caused by the surgical dissection, fixation techniques, and perioperative management [25]. Most of the studies suggest that the outcome after a Lisfranc lesion improves as the quality of the reduction improves [3, 4, 6, 8, 10, 16, 30–39]. However, an anatomical reduction does not guarantee a good outcome [6, 10, 16, 29, 33, 36–40].

Like other authors [31, 41, 42], we consider that even an almost anatomical reduction is acceptable and predicts a favorable outcome for in our series we obtained four anatomical reductions and one almost anatomical reduction without evidence of significant clinical differences among them (clinical-radiological dissociation). Anyway, we admit the number of cases is not statistically significant.

Perugia et al. [42] reported an AOFAS score of 81 points with no significant differences in the results between anatomical and almost anatomical reductions. In our series, we obtained an average of 96 points in the same scale.

When the Lisfranc fracture dislocation is secondary to some low-energy trauma and the soft tissue lesion is mild, an anatomical or almost anatomical reduction may be achieved with a percutaneous reduction and screw fixation [26].

In agreement with this, Alberta et al. [43] believe that transarticular screw fixation is the preferred option for most of the orthopedic surgeons to fix the medial and middle columns. This type of stabilization provides rigid fixation and may be placed percutaneously. However, some disadvantages have been identified for it causes damage in the hyaline cartilage which is supposed to be preserved.

Schepers et al. [12] found that the articular damage is considerable, involving about 2% to 7.6% of the joint area. Apart from transarticular screws, an extra-articular bridge plate may be inserted, which provides similar stability [43].

Apart from preventing articular cartilage damage, plates also have other advantages when compared to transarticular screws. If the screws break, distal threads are typically intra-articular and may increase the chondral damage with movement. Also, they are difficult to remove without additional significant involvement.

If a plate breaks in the joint, joint movement may occur and it is not necessary to remove the material. If the screws inserted through the plate are broken, the distal threads may be left in the metatarsals or cuneiforms with no risk of more irritation of soft tissues or joint damage; or else they are easily accessible without contacting the joint, by specific instruments to remove screws [43].

Therefore, the morbidity risk would be lower in plate fixation, and less associated risk for both mobility and weight load can be achieved early on after surgery leading to less joint rigidity, muscle atrophy, and disuse osteopenia.

The disadvantage of plates as compared to screws is that plates can not be inserted percutaneously, and so an open reduction is required in patients who might simply need a closed reduction.

However, many surgeons typically perform an open reduction to eliminate any interposed intra-articular tissue and confirm that the reduction is anatomical due to direct exposure [3, 10, 37, 43, 44]. Plates can not be placed percutaneously; however, they can be inserted smoothly by a medial approach and a minimally invasive procedure using the proximal portal to also insert the Lisfranc screw (C1-M2). Moreover, an open reduction is not free from complications [33]. Several incisions are typically needed, which increases the risk of skin necrosis and delayed wound healing [26, 45].

Also, additional soft tissue dissection, including the potential lesion of the distal insertion of the anterior tibial tendon, may cause increased morbidity [43].

Plates are more likely to cause soft tissue irritation due to their prominence: this irritation is usually transient for many surgeons remove the osteosynthesis material routinely 3 to 4 months after the fixation. We routinely remove the plates as scheduled four months after the procedure. The development of low-profile plates, such as the plates used in this study, has successfully led to a decreased incidence of periarticular soft tissue irritation associated with the osteosynthesis material.

As reported by Alberta et al. [43] plate insertion takes longer in the OR, and more dissection of the first cuneiform is required. For a minimally invasive procedure, a shorter time and less dissection are needed. Moreover, locking plates offer a feasible option for a more stable fixation [46, 47]. For this reason we were able to perform reduced stabilization with fewer screws at both sides of the joint. Plate placement where plantar tension of TNT joints is located would be ideal from the biomechanical viewpoint; however, it is not clinically applicable in acute Lisfranc lesions^3^ [43].

Marks et al. [48] performed a study in cadavers where they compared stabilization with a plantar plate plus Lisfranc screw versus transarticular screws C1-M1, C1-M2, and C1-C2. Plate fixation proved to be stronger and exhibit less secondary displacement after weight load application. To date, we do not know whether there are any biomechanical studies available comparing medial plates with plantar plates or with transarticular fixation; but as our study suggests medial plates plus a C1-M2 stabilization screw, although biomechanically weaker than plantar plates, may be comparable to the stabilization achieved with transarticular screws, and the clinical results are similar.

Thordarson and Hurvitz [44] obtained four nonanatomical reductions in six Lisfranc lesions managed with closed reduction and concluded that a nonanatomical reduction may play a significant role in increased arthrosis changes [10, 49, 50]. However, in spite of an anatomical reduction, the presence of posttraumatic arthrosis changes is still the most common complication after Lisfranc lesions [16, 51, 52].

It is estimated that the number of patients who develop arthrosis after a tarsometatarsal lesion is roughly 20 to 50% [10, 50, 53, 54]. These changes may be slight degenerative or even present complete loss of the articular space [13]. Fortunately, most of these findings are progressive and exhibit minimum function loss [55].

As reported by Myerson [3] “it is interesting to recognize the lack of association between the level of arthritis and symptoms.”

In our series, we have not evidenced arthrosis-related changes or complete loss of the joint space; this may be related to the short follow-up period and, to a lesser extent, to the quality of the reduction.

The surgical technique described in this report including patients with closed, low-energy, partial, unstable Lisfranc lesions is novel; it is characterized by the use of implants and a novel approach to reduce joint and soft tissue damage.

We performed a closed reduction and minimally invasive stabilization with a bridge plate and a screw after achieving a closed anatomical reduction. Rigid internal fixation was achieved in all cases. In spite of the small sample, the initial results do not show intraoperative or postoperative complications.

However, certain strengths are identified: the population characteristics (homogeneous population, low-energy lesions involving the medial and middle columns) and prospective design; the same surgeon experienced in complex reconstructive procedures.

The technique described in this case report series is a valid surgical procedure applicable to selected patients and provides stable internal fixation and significant reduction of the risk of iatrogenic articular involvement related to percutaneous techniques.

## Figures and Tables

**Table 1:** 

*N*	Name	Age	Myerson clas.	Asoc. fx.	FU (m)	Reduction	AOFAS sc.	VAS	WB (d)	TS (min)
1	A, B.	25	B2	C1	20	Anatomical	95	1	40	49
2	I, E.	41	B2	M2	21	Anatomical	98	2	44	52
3	SF, M	38	B2	M3	19	Anatomical	90	3	46	46
4	M, A	41	B2	No	19	Almost anatomical	100	0	41	40
5	M, S	67	B2	No	18	Anatomical	97	1	41	50

		42.4			19.4		96	1.4	42.4	47.4

Note: fx.: fracture; C: cuneiform; M: metatarsal; FU: follow-up; (m): months; sc.: score; VAS: Visual Analogic Scales; WB: weight bearing; (d): days; TS: time of surgery.
